# Characterization and Optimization of the Seeding Process of Adipose Stem Cells on the Polycaprolactone Scaffolds

**DOI:** 10.1155/2019/1201927

**Published:** 2019-02-20

**Authors:** Agata Kurzyk, Barbara Ostrowska, Wojciech Święszkowski, Zygmunt Pojda

**Affiliations:** ^1^Department of Regenerative Medicine, Maria Sklodowska Curie Institute-Oncology Center, Roentgena 5, 02-781 Warsaw, Poland; ^2^Materials Design Division, Faculty of Material Science and Engineering, Warsaw University of Technology, Woloska 141, 02-507 Warsaw, Poland

## Abstract

The purpose of the current study was to evaluate the usefulness of adipose-derived stem cells (ASCs) for bone injury therapy. Lipoaspirates were collected from the abdomen regions of 17 healthy female donors (mean age 49 ± 6 years) using Coleman technique or Body-jet liposuction. In the present study, the primary objective was the *in vitro* characteristics of human ASCs. The secondary objective was the optimization of the cell seeding process on 3D-printed scaffolds using polycaprolactone (PCL) or polycaprolactone covered with tricalcium phosphate (PCL + 5% TCP). Biological evaluation of human ASC showed high efficiency of isolation obtaining a satisfying amount of homogeneous cell populations. Results suggest that ASCs can be cultured *in vitro* for a long time without impairing their proliferative capacity. Growth kinetics shows that the highest number of cells can be achieved in passage 5 and after the 16^th^ passage; there is a significant decrease of cell numbers and their proliferative potential. The percentage of colony forming units from the adipose stem cells is 8% ± 0.63% (*p* < 0.05). It was observed that the accumulation of calcium phosphate in the cells *in vitro*, marked with Alizarin Red S, was increased along with the next passage. Analysis of key parameters critically related to the cell seeding process shows that volume of cell suspension and propagation time greatly improve the efficiency of seeding both in PCL and PCL + 5% TCP scaffolds. The cell seeding efficiency did differ significantly between scaffold materials and cell seeding methods (*p* < 0.001). Increased seeding efficiency was observed when using the saturation of cell suspension into scaffolds with additional incubation. Alkaline phosphatase level production in PCL + 5% TCP scaffold was better than in PCL-only scaffold. The study results can be used for the optimization of the seeding process and quantification methods determining the successful implementation of the preclinical model study in the future tissue engineering strategies.

## 1. Introduction

Regenerating or replacing bone defects is an important research field in tissue engineering. Current methods for surgical treatment of fractures and bone defects primarily use metal implants, and autologous and allogeneic bone grafts still represent the gold standard for bone repair. Development of new treatments is mainly focused on the tissue engineering strategies that include stem cells, bioactive signals, and appropriate scaffold support. Mesenchymal stem cells derived from adipose tissue are promising cell source for bone lesion repair [1]. This is important for the optimization of methods aimed at isolation, characterization, expansion, and evaluation of differentiation potential [2]. These parameters ensure the quality of stem cells and the safety of their use. Harvesting procedure, tissue site, age, obesity, and related-chronic diseases may influence cell yields from adipose tissue. ASCs can be isolated from adipose tissue during previous surgical resection or liposuction [2]. Several approaches for ASC isolation have been reported [3, 4], but data comparing the efficacy of various methods are still not available; therefore, no standardized method exists. The protocol described in 2001 by Zuk et al. is still considered as the most widely used method for ASC isolation, based on digestion with collagenase [5]. There are conflicting reports on the effect of donor age on adipose human mesenchymal stem cells [6–8]. By contrast with bone marrow-derived MSCs, the number of ASCs in adipose tissue does not decrease with age [7, 8] even if their clonogenic and proliferative potential gradually declines. Numerous studies have reported that ASCs isolated from old individuals have reduced function and adipogenic potential compared to ASCs from young subjects [9–11]. The growth rate of ASCs has been reported also to be higher in younger patients (25–30 years old) than in older patients [12]. Nevertheless, adipose tissue displays a significant heterogeneity in terms of stem cell yield, proliferation, and differentiation capacity. Therefore, the primary objective of the present study is aimed at characterizing ASCs from the abdomen regions of 17 healthy female donors (mean age 49 ± 6 years) in order to investigate yield of cell number of stromal vascular fraction (SVF), proliferation, and potential of osteogenic differentiation and for possible evaluation of the usefulness of adipose stem cells (ASC) passage 3 for the construction of polymer-cell scaffolds.

Optimization of cell seeding on polymer scaffolds is essential for the successful *in vitro* cultivation of functional tissue constructs [1]. General seeding requirements for 3D scaffolds include high yield, to maximize the utilization of donor cells; high kinetic rate, to minimize the time in suspension culture for anchorage-dependent and shear-sensitive cells; spatially uniform distribution of attached cells, to provide a basis for uniform tissue regeneration; and high initial construct cellularity, to enhance the rate of tissue development. Additional seeding requirements may depend on cell type and tissue engineering application. The applied seeding technique therefore needs to be simple and easy to use, repeatable, and time-efficient [13]. Besides the individual effect, there is many crucial factors exert on the seeding process and also their interactions play a major role in determining outcome parameters [14–16]. It has been shown that changes in the scaffold porosity significantly affect the mesenchymal stem cell seeding efficiency in 3D-engineered bone scaffolds. In addition, many researches have shown that the initial seeding density did not significantly affect cell seeding efficiency, but the seeding time and the volume of seeding. The biological mechanisms responsible for the regenerative potential of stem cells are still not fully understood.

However, still little is known how to effectively optimize the seeding of scaffolds for bone regeneration. Thus, there is still a need for a simple effective way of improving cell seeding efficiency on scaffolds. Therefore, the secondary objective of this work was to select parameters that can play a key role in effectively seeding ASCs into polycaprolactone scaffolds. Our results may also provide information how seeding method selection affects cell distribution and how modification of scaffolds may influence the seeding efficiency.

## 2. Materials and Methods

### 2.1. Experimental Design

Adipose tissue was collected from raw human lipoaspirates according to Coleman technique or Body-jet liposuction. Lipoaspirates were obtained from the abdomen regions of 17 healthy female donors. The age range of the patients was 40–59 years (mean age 49 ± 6 years). All patients consented written forms for the use of the material (lipoaspirate) for the tests, in accordance with the standards of the International Ethical Committee.

### 2.2. Isolation and Culture of ASCs

Briefly, raw lipoaspirates were washed extensively with sterile phosphate-buffered saline (PBS) (Gibco®, Life Technologies, USA) to eliminate contaminating debris and red blood cells. Washed aspirates were treated with 0.075% collagenase (*Clostridium histolyticum*; type I; Sigma-Aldrich, St. Louis, MO, USA) in PBS for 60 min at 37°C with gentle agitation. The collagenase was inactivated with 10% fetal bovine serum (FBS, Gibco®, USA), and the infranatant centrifuged (400 ×g, 10 min, 23°C) until phase separation. To filter out larger tissue particles, the stromal vascular fraction (SVF) pellet was resuspended and passed through a 100 *μ*m filter placed atop a new 50 ml centrifuge tube to facilitate gravitational separation. The filtrate was centrifuged at 400 ×g for 10 min to obtain a high-density SVF pellet containing ASCs, which was then cultured in tissue-culture-treated culture dishes in a medium comprising an equal volume of low-glucose Dulbecco's modified Eagle's medium (DMEM, Gibco®, USA) and fetal bovine serum (FBS) and incubated in a humidified atmosphere at 37°C and 5% CO_2_. Media were replaced twice per week, and cells were passaged on approaching 80–90% confluence, using 0.25% trypsin-EDTA solution (Invitrogen, USA). Routine tests were performed before ASC preparation and after preparation, including sterility control (BACTEC, BacT/Alert, Becton Dickinson, blood agar, Columbia agar, and BHI agar), cell enumeration (Bürker chamber), and viability analysis (fluorescence microscopy, acridine orange plus ethidium bromide staining).

### 2.3. Flow Cytometric Analyses of ASC Surface Markers

ASCs in passage 3 were harvested, enumerated, and incubated for 20 min with the following specific fluorescent conjugated antibodies including anti-CD29-PE, anti-CD34-PE, anti-CD45-FITC, anti-CD73PE, anti-CD90-PE, anti-CD105-PE, anti-CXCR-4-PE, and lin1-PerCP (anti-CD3-FITC, anti-CD14-FITC, anti-CD16-FITC, anti-CD19-FITC, anti-CD20-FITC, and anti-CD56-FITC). Control staining with directly labeled, isotype matched monoclonal antibodies was included in all fluorescence-activated cell sorting (FACS) experiment control (*γ*-PE, *γ*-PerCP, and *γ*-PE CD105) (BD, USA). Flow cytometry was performed using a Calibur flow cytometer (BD, USA) and CellQuest Pro software (BD, USA). Data are represented as mean values (*M*) ± standard deviation of the mean (SD) for 17 individual donors.

### 2.4. Morphology and Multilineage Differentiation Potential of hASCs

The potential of ASC to differentiate into osteoblasts and adipocytes was confirmed in monolayer culture. To induce adipogenic differentiation, cells were cultured in MesenCult™ adipogenic stimulatory supplements (human) (STEMCELL Technologies, Canada) supplemented with MesenCult® MSC basal medium (STEMCELL Technologies, Canada). To induce osteogenic differentiation, we used MesenCult™ osteogenic stimulatory supplements (human) (STEMCELL Technologies, Canada) in the osteogenic medium containing 10^−4^ M dexamethasone (STEMCELL Technologies, Canada), 1 M *β*-glycerophosphate (STEMCELL Technologies, Canada), and 10 mg/ml ascorbic acid (STEMCELL Technologies, Canada). Media from both cultures were replaced every 3 d for 21 d in total. The differentiation potential for adipogenesis and the formation of intracellular lipid droplets were assessed by Oil Red O staining after fixation in 10% formalin. The differentiation potential for osteogenesis was assessed by Alizarin Red S (ARS) staining after fixation in 10% formalin. To induce chondrogenic differentiation, we used a chondrogenic medium (LONZA, Switzerland). For histological analysis, pellets were embedded in paraffin and sectioned. Chondrogenic differentiation was assessed by Masson trichrome staining. Morphology and differentiation potential of ASCs are represented for one individual donor from three independent donors.

### 2.5. Growth Kinetics

To investigate the growth pattern of ASCs, SVF cells were seeded at a density 1–2 × 10^6^ cells/T25. The cells were grown in DMEM containing 10% FBS at 37°C, 5% CO_2_, and 95% humidity. After approaching 80% confluence, the cells were passaged. After primary culture, the passaged cells were plated on a T25 culture bottle at a density 0.3 × 10^6^ cells/T25 and their reproductive rate was recorded. After each passage, culture media were replenished every 3 days until the end of experiment. Cells were then trypsinized and enumerated. Cells were passaged; after which, they were unable to undergo further cell division.

### 2.6. Clonogenic Potential

The SVF cells isolated from human lipoaspirates were evaluated for their clonogenic ability by using colony-forming (CFU-F) assay. The cells were seeded at a low density (40 cells/cm^2^) and cultured in DMEM containing 10% FBS for 6–7 days. Colonies were stained with Giemsa stain, and those comprising >50 cells were enumerated manually using a light microscope. The colony potential was compared by calculating the percentage of cell-forming colonies/number of cells seeded × 100. Clonogenic potential is a representative for 6 individual donors.

### 2.7. Scaffold Manufacturing

Poly(*ε*-caprolactone) scaffolds with custom geometry and controlled internal architecture were developed using fused deposition modeling at Faculty of Materials Science and Engineering, Warsaw University of Technology. A composite comprising PCL Mn 80.000 (Sigma-Aldrich) and *β*-TCP (Progentix, catalog no. P08004C) was used. Cylindrical scaffolds (6 mm diameter and 4 mm height) with three-dimensional orthogonal periodic porous architecture were designed using SOLIDWORKS 3D CAD design software. Layer-by-layer printed microfibrous scaffolds were characterized by the following theoretical parameters: fiber diameter (D1), 330 *μ*m; spacing between fibers in the same layer (D2), 420 *μ*m; layer thickness (D3), 240 *μ*m; layer deposition angle, 0/60/120 the same layer (D2), 420 *μ*m; layer thickness (D3), 240 *μ*m; and layer deposition angle, 0/60/120.

### 2.8. Sterilization of Scaffolds

Scaffolds (PCL, PCL + 5% TCP) were irradiated at Gamma Chamber 5000 on the dose rate of 8.80 kGy/h in air. The total delivered radiation dose was 25 kGy. Sterilization was performed at the Institute of Chemistry and Nuclear Technology, Warsaw University of Technology. Samples for sterilization were delivered in sealed and sterile containers.

### 2.9. Fixing of Cell-Seeded Scaffolds

Cell-scaffold constructs were fixed in 1.5% glutaraldehyde containing a 0.1 M sodium cacodylate buffer (pH 7.3), rinsed twice with PBS, and left to dry at room temperature.

## 3. Comparison of Different Strategies for *In Vitro* Seeding of Scaffolds

### 3.1. Basic Static Cell Seeding Methods

**Figure 1:**
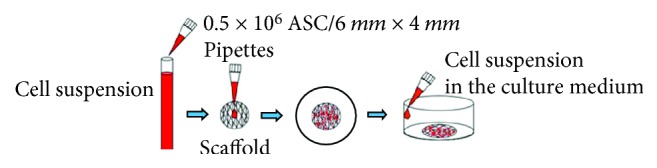
The static method of cell seeding into scaffold. Cell suspension is pipetted directly into the central surface of the scaffold.

Sterilized scaffolds PCL and PCL + 5% TCP were placed in 24-well tissue culture plates. Culture-expanded human ASCs (passage 3) were suspended in the DMEM medium containing 20% FBS, using 0.5 × 10^6^ cells in three variants of volumes: 50 *μ*l, 35 *μ*l, and 20 *μ*l. The cell suspension was added on the top of each scaffold and pipetted in and out, to enhance even distribution of cells within the scaffolds. The prepared plate was gently transferred to an incubator under conditions of 37°C, 98% humidity, and 5% CO_2_ (Figure 1). The medium was changed every three days. Cell-seeded scaffolds were cultured for 21 and 42 days. After this time, cell-seeded scaffolds were fixed and analyzed using a visible light microscope. The process was performed 6 times for each group. To determine cell seeding efficiency, cell numbers in randomly selected scaffolds were determined after 3, 7, and 21 days of incubation using an MTS assay (CellTiter 96® AQueous One Solution Cell Proliferation, Promega, Madison, WI, USA) (*n* = 3). Test MTS was repeated 3 times, from 3 independent donors.

### 3.2. Saturation of Cell Suspension into Scaffolds Combined with Additional Incubation

**Figure 2:**
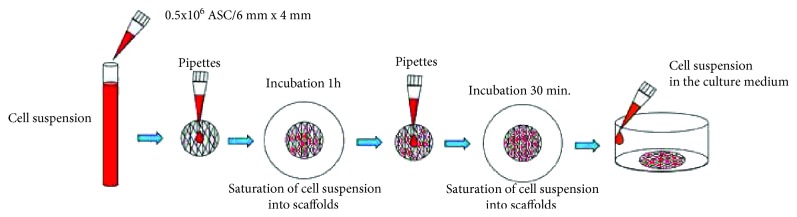
The method of saturation of cell suspension into scaffolds with additional incubation.

Sterilized scaffolds (PCL and PCL + 5% TCP) were placed in 24-well tissue culture plates. Culture-expanded ASCs (passage 3) were suspended in the DMEM medium containing 20% FBS, using 0.5 × 10^6^ cells, in two volumes: (1) 50 *μ*l and (2) 35 *μ*l. At the beginning, half volume of total cell suspension (1) 25 *μ*l from 50 *μ*l and (2) 17.5 *μ*l from 35 *μ*l was added on the top of each scaffold and pipetted in and out, to enhance even distribution of cells within the scaffolds. The prepared plate was gently transferred to an incubator under conditions of 37°C, 98% humidity, and 5% CO_2_. After 60 minutes in the incubator, the remainder of volume (1) 25 *μ*l and (2) 17.5 *μ*l was added to the scaffolds, which were subsequently stored in the incubator for 30 minutes. After this time, the scaffolds were placed in different, sterile tissue culture wells, one scaffold per well. Fresh medium (basic medium DMEM with 20% FBS) was added to the cell-seeded scaffolds (Figure 2). The medium was changed every three days. Cell-seeded scaffolds were cultured for 21 days. After this time, cell-seeded scaffolds were fixed and microscope-analyzed. To determine cell seeding efficiency, cell numbers on randomly selected scaffolds were determined after 3, 7, and 21 days of incubation using and MTS assay (CellTiter 96® AQueous One Solution Cell Proliferation, Promega, Madison, WI, USA). Test MTS was repeated 3 times, from 3 independent donors.

### 3.3. Optimization of Density of Seeding Cells into Scaffolds

Sterilized scaffolds (PCL and PCL + 5% TCP) were placed in 24-well tissue culture plates. Culture-expanded human ASCs (passage 3) were suspended in the DMEM medium containing 20% FBS, using three densities of cell suspensions: 0.5 × 10^6^, 0.9 × 10^6^, and 1.5 × 10^6^. The medium was changed every three days. Cell-seeded scaffolds were cultured for 3, 7, and 21 days. The process was performed 7 times.

### 3.4. Response Measurements and Data Analysis

The response measurements are based on the metabolic activity and alkaline phosphatase activity of the cells in the seeded scaffolds. In addition, the cell spatial distribution was also evaluated for the cell seeding on 3D scaffolds.

### 3.5. Cell Metabolic Activity Analysis

To determine the cell seeding efficiency, cell numbers on some seeded scaffolds were determined after 1, 3, 7, and 21 days of incubation, using the 3-(4.5-dimethylthiazol-2-yl)-5-(3-carboxymethoxyphenyl)-2-(4 sulfophenyl)-2H-tetrazolium (MTS) assay (CellTiter 96® AQueous One Solution Cell Proliferation, Promega, Madison, WI, USA). The MTS assay (CellTiter 96® AQueous One Solution Cell Proliferation Assay, Promega Corporation, Fitchburg, WI, USA) was used to measure the metabolic activity of the cells. Twenty microliters of MTS was added to 100 *μ*l of fresh culture medium per well. The plates were incubated for 4 h at 37°C and 5% CO_2_. Thereafter, the optical density was measured spectrophotometrically in 100 *μ*l per well, using a multimode microplate reader (Synergy HT; BioTek, Winooski, VT, USA) at 490 nm. At this point, the MTS assay was performed to determine the total cell numbers for each scaffold, as described above. See MTS standard curve in Supplementary Materials.

### 3.6. Alkaline Phosphatase Activity Assay

ALP activity of the cells was measured using a Thermo Fisher Scientific PNPP phosphate substrate kit (Pierce, USA); P-nitrophenyl phosphate (pNPP) was used as the phosphatase substrate, which yields a yellow coloration (*λ*_max_ = 405 nm) when dephosphorylated by ALP. The activity of this enzyme was monitored on days 7 and 14. First, the medium was aspirated from the wells and 100 *μ*l of pNPP reagent was added to each well with a scaffold sample. Thereafter, the plates were incubated for 30 min at 37°C and 5% CO_2_ until sufficient coloration developed, and then, pNPP reagent was transferred into new wells. The reaction was terminated by adding 50 *μ*l of 2 N NaOH to each well. The optical density was recorded using a microplate reader, at 405 nm.

### 3.7. Evaluation of Cell Growth after Cell Seeding Scaffolds

The prepared scaffold composites were examined by the CKX41 inverted microscope, confocal microscope, and SEM. The morphology and structure of the scaffolds were studied at 42 days following cell seeding using a scanning electron microscope (SEM, SU8000, Hitachi, Tokyo, Japan). The diameters of 50 randomly selected fibers were measured using image analysis software (ImageJ, National Institute of Health, Bethesda, MD, USA). The prepared and fixed cell-seeded scaffolds PCL and PCL + 5% TCP were examined by SEM at Faculty of Materials Science and Engineering, Warsaw University of Technology.

### 3.8. DAPI Staining Test

Stem cell-seeded scaffolds were fixed with 4% paraformaldehyde at 4°C for 30 min on days 1, 3, and 5 after cell seeding. Then, samples were washed twice with PBS, incubated with 4′, 6-diamidino-2-phenylindole (DAPI) (Sigma Chemical Co., USA) for 30 seconds to label nuclei of the cells, and rinsed twice with PBS. The immunofluorescence images were obtained by using a fluorescence microscope. Experiment was repeated 3 times, from 3 independent donors.

### 3.9. Statistics

The statistical analysis of the material was carried out using statistical tests of the STATISTICA v. 10 package by StatSoft Polska (comparison of the seeding methods on PCL and PCL + 5% TCP scaffolds) and the statistical program R version 3.4.3 (2017). The Shapiro-Wilk test was performed to assess normality; hypothesis testing was performed using the Benjamini-Hochberg test, and analysis of variance (ANOVA), followed by a Tukey test (for variables with normal distribution) for post hoc analysis or a Kruskal-Wallis test with Dunn's test (nonparametric) for post hoc analysis, was performed. Variables are presented as mean (*M*) ± standard deviation of the mean values (SD). A *p* value less than 0.05 indicated statistical significance, and additional significance was indicated with ^∗^ *p* < 0.05, ^∗∗^ *p* < 0.01, and ^∗∗∗^ *p* < 0.001.

## 4. Results

### 4.1. Flow Cytometry

**Figure 3:**
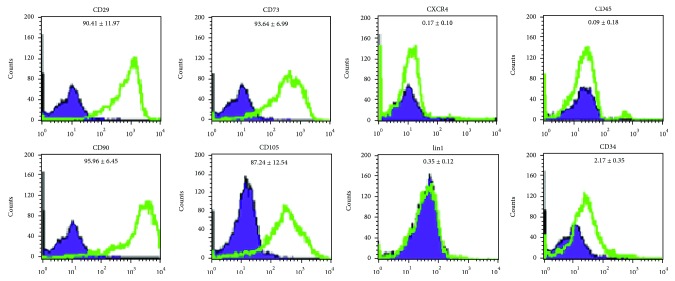
Histograms show results of ASC staining for indicated surface markers from one representative donor, passage 3. Cell counts are indicated on the *y*-axis and fluorescence intensity on the *x*-axis. The percentages of ASCs positively stained are indicated in each panel. Violet: isotype controls; green: antigen-specific antibodies.

Cultures of ASCs at passage 3 were analyzed for the expression of cell surface markers. ASCs were negative for the hematopoietic lineage marker CD45, on average 0.09% ± 0.18% CD45, 2.17% ± 0.35% CD34, and 0.35% ± 0.12% lin1. ASCs were positive for CD29, CD90, and CD105, on average 90.41% ± 11.97% CD29, 95.96% ± 5.45% CD90, and 87.24% ± 12.54% CD105. Representative histograms show results of ASC staining for indicated surface markers from one representative donor (Figure 3).

### 4.2. Morphology and Multineage Differentiation Potential of ASCs from Human Lipoaspirate

**Figure 4:**
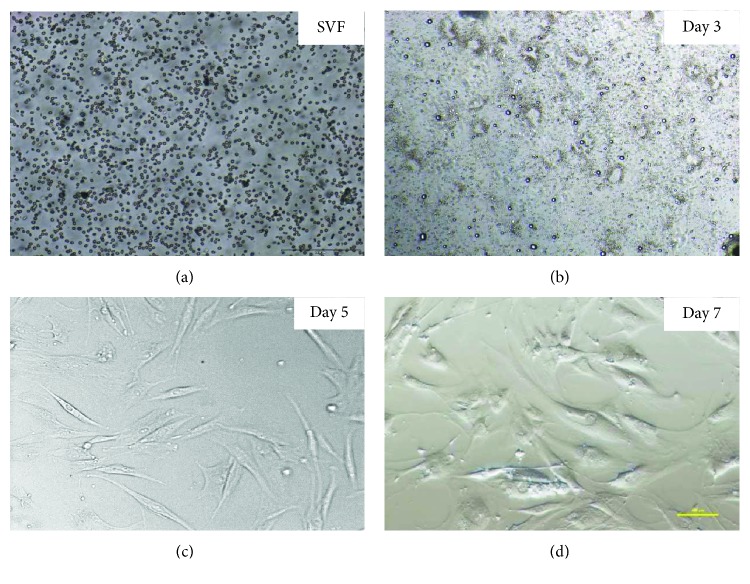
The representative images of human ASCs after fresh isolation, 3, 5, and 7 days of 2D culture. Representative images are shown at 4x magnification. Scale bars represent 100 *μ*m (Olympus CKX41). The representative images are from one donor.

The shape of the isolated cells was initially round (Figure 4(a)) and became polygonal or spindle-shaped thereafter (ASC cells) (Figures 4(b)–4(d)). Attachment of spindle-shaped cells to tissue culture plastic flask was observed after 5 days of ASC culture (Figure 4(c)). After 7 days, spindle-shaped cells reached above 80% (Figure 4(d)). Morphology of cells changed gradually with passage number. Cells become more flat shape with increasing passage number.

### 4.3. Proliferation and Growth Kinetics of ASCs

**Figure 5:**
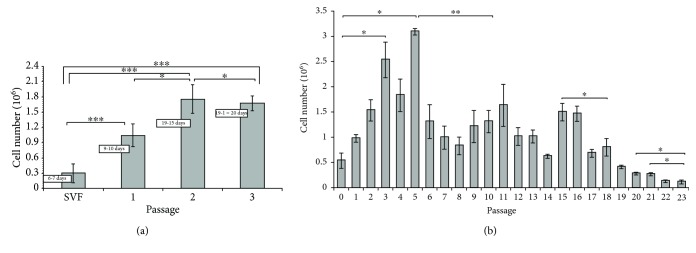
The number of human adipose stem cells obtained in individual passages. (a) From isolation SVF to passage 3. *M* ± SD (*n* = 5). (b) From passage 0 to passage 23. *M* ± SD (*n* = 3).

**Figure 6:**
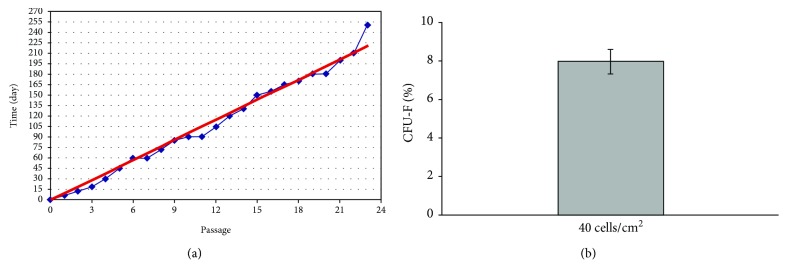
(a) The association between the duration of adipose stem cell cultures and individual passages in cells obtained from human adipose tissue (*n* = 5). (b) Clonal growth test (CFU-F) of stromal vascular fraction cells obtained from human adipose tissue. *M* ± SD (*n* = 6).

ASCs were obtained from abdomen tissue from 17 female donors over a broad age range (49 ± 6 years). ASCs were successfully obtained from all donors. Yield of SVF cells was average 0.36 × 10^6^ per milliliter of lipoaspirate, viability average 86%. Characterization of donors and number of SVF are presented in Supplementary Materials (Table 1). The growth curve (Figure 5(a)) describes the kinetics of cell proliferation in primary culture (SVF) and the first three secondary cultures (ASC). Differences between individual passages are statistically significant. The maximum ASC proliferation capacity was observed in passages from 2 to 5 (^∗^ *p* < 0.05). A significant decrease of cell number and their proliferative potential is occurred after the 6^th^ to 16^th^ passages. Significant differences are also observed between passages 20 and 23 (^∗^ *p* < 0.05). Results of average number of ASCs are presented in Supplementary Materials (Table 2). Growth kinetics is represented for 5 individual donors (Figure 5(a), from SVF to passage 3) and for 3 individual donors (Figure 5(b), from SVF to passage 23). Analyzing the propagation time of culture of human ASCs in terms to particular passages, we observed that the time of expansion of SVF cells from primary culture to passage 1 was on average 6 days. Passage 3 was obtained after 15–19 days, and passage 6 was obtained after 60 days (Figure 6(a)). CFU-F assay is a suitable tool for evaluating the proliferation and clonogenic capacity of the SVF expanded in culture. Giemsa staining showed that human SVF cells are able to form colonies from a single progenitor cell. It was observed 8% ± 0.63%. CFU-F colonies at a density of 40 cells/cm^2^ were observed as shown in Figure 6(b).

### 4.4. Multipotency: Osteogenesis, Chondrogenesis, and Adipogenesis

**Figure 7:**
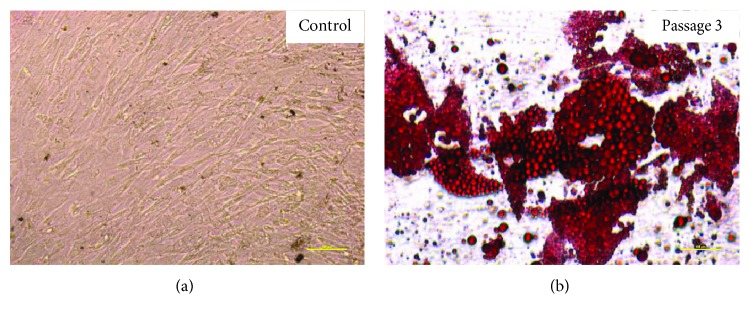
Oil Red O staining of human adipose stem cells after passage 3. (a) Control, undifferentiated adipose stem cells. (b) Adipogenic differentiation. Representative images are shown at 10x magnification. Scale bars represent 100 *μ*m. The representative images are from one independent donor.

**Figure 8:**
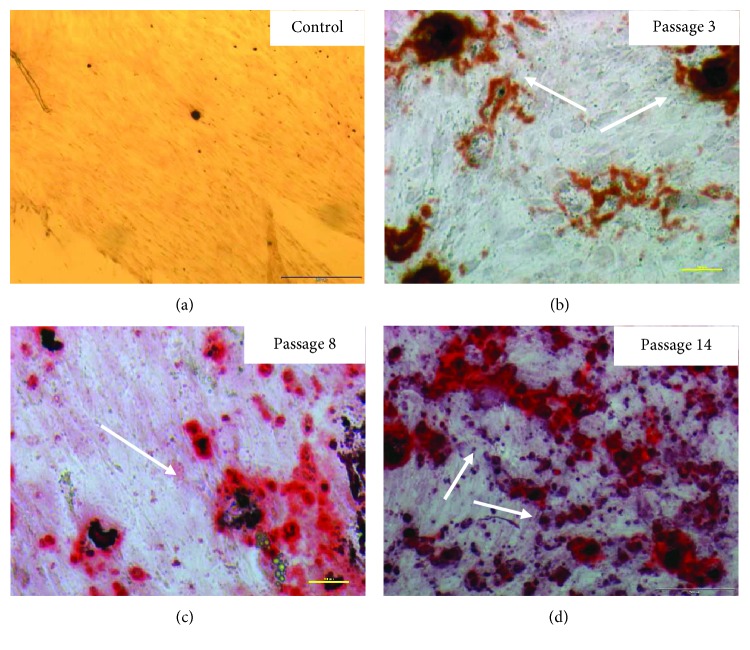
Alizarin Red staining and mineralization assay of human adipose stem cells after 3, 8, and 14 passages. (a) Control, undifferentiated ASCs. (b, c, d) Osteogenic differentiation. Representative images are shown at 10x magnification. Scale bars represent 100 *μ*m. The representative images are from one independent donor. Calcium deposits are indicated with white arrows.

**Figure 9:**
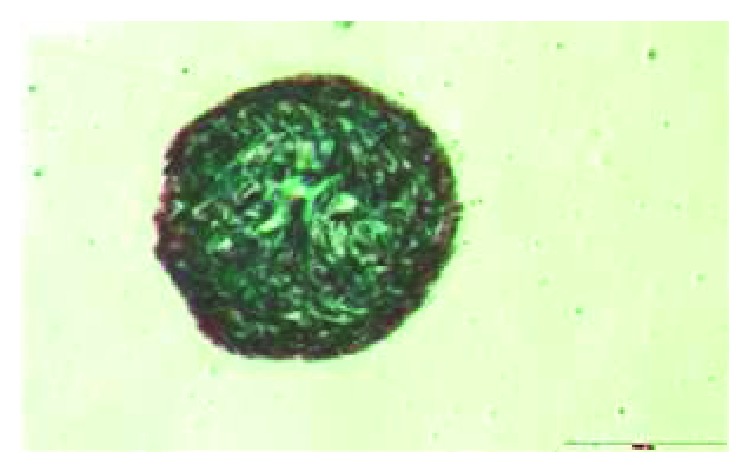
Chondrogenic differentiation of the human adipose stem cells. After 3 weeks of chondrogenic induction, the pellet was observed. Pellets were stained with Masson's trichrome stain. Representative images are shown at 4x magnification. Scale bars: 200 *μ*m. The representative images are from one independent donor.

Human ASCs showed ability to differentiate to osteocyte, adipocyte, and chondrocyte. Upon applying adipogenic differentiation, the cells showed accumulated intracellular lipid droplet as revealed by Oil Red O staining (Figure 7). Osteogenic differentiation displayed extracellular calcium precipitates, which were identified by Alizarin Red staining (Figure 8) and chondrogenic differentiation demonstrated by Masson's trichrome staining (Figure 9). Microscopic observation indicates that these cells can differentiate into adipocytes, osteoblast, and chondroblast. Control cultures were added to the experiment using basic culture medium, where there was no cell differentiation (Figures 7(a) and 8(a)). Moreover, it was observed that osteogenic potential increased with increasing passage.

### 4.5. Effect Volume of Cell Suspension for Efficiency of Seeding

**Figure 10:**
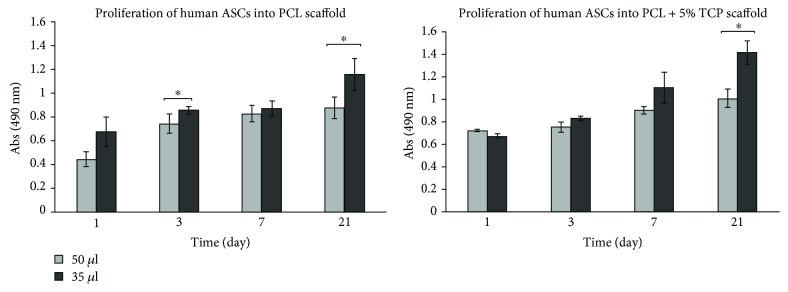
Seeding efficiency of human ASCs into PCL and PCL + 5% TCP scaffolds after 1, 3, 7, and 21 days. All scaffolds were seeded with 0.5 × 10^6^ ASCs, passage 3. Volume of cell suspension: 50 *μ*l and 35 *μ*l (*n* = 3 for each group). Significance: ^∗^ *p* < 0.05.

To evaluate the effect of cell seeding and growth on 3D tissue engineering scaffolds which we placed into the well *in vitro*, cell metabolism was analyzed over a three-week period (days 1, 3, 7, and 21). Analysis of the proliferation of human ASCs suspended in the seeding volume of 50 *μ*l and 35 *μ*l showed statistically significant differences after 3 and 21 days of culture into PCL (^∗^ *p* < 0.05) and after day 21 of culture for PCL + 5% TCP scaffold. Results shows that cell seeding efficiency increased when using a seeding volume of 35 *μ*l. We also observed a greater cell activity on the scaffolds on day 21 (Figure 10). Our results may suggest that, except volume cell seeding, also propagation time plays an important role in efficiency of seeding. See raw data in Supplementary Materials (Table 3).

### 4.6. Comparison of Two Different Strategies for *In Vitro* Seeding of Human ASC of PCL and PCL + 5% TCP Scaffolds

**Figure 11:**
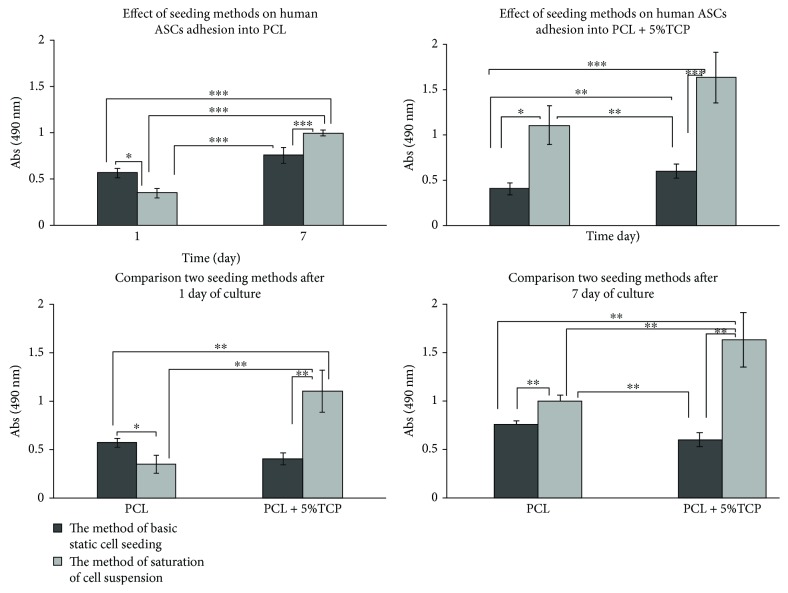
Metabolic activity of the human adipose stem cells measured by MTS assay into PCL and PCL + 5% TCP scaffolds (*n* = 3). Notes: statistical analysis: ^∗^ *p* < 0.05, ^∗∗^ *p* < 0.01, ^∗∗∗^ *p* < 0.001.

To compare the effect of two methods to cell seeding PCL and PCL + 5% TCP, cell metabolism was analyzed over a week period (1 and 7 days). Results indicate that ASC cells attached and proliferated on PCL and PCL + 5% TCP scaffolds cultured in a basic medium. We observed significant differences between two methods used (^∗∗^ *p* < 0.001). See raw data in Supplementary Materials (Table 4). Increased seeding efficiency was observed when using the saturation of cell suspension into scaffolds with additional incubation. Basic static cell seeding method did not generate this level of seeding efficiency.  Figure 11 contains an assessment of the statistical significance between the method of basic static ASC cell seeding and the method of saturation of cell suspension, respectively. Results implying that the selection of the method of cell seeding can significantly increase seeding efficiency. Data presents a higher level of ASC proliferation into PCL + 5% TCP than PCL scaffold.

### 4.7. Optimization of Density of Seeding Cells into Scaffolds

**Figure 12:**
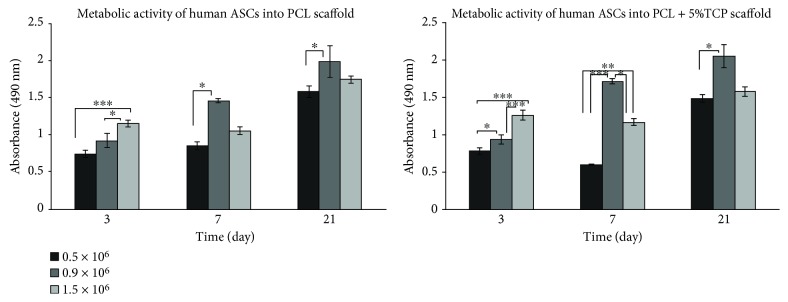
Metabolic activity of the human adipose stem cells measured by MTS assay into PCL and PCL + 5% TCP scaffolds (*n* = 3). Statistical analysis: ^∗^ *p* < 0.05, ^∗∗^ *p* < 0.01, and ^∗∗∗^ *p* < 0.001.

The level of metabolic activity measured by the MTS test for human ASCs placed on PCL and PCL + 5% TCP significantly increased in cells after 3, 7, and 21 days of culture using the initial cell number equal to 0.9 × 10^6^ (6 mm × 4 mm of the scaffold area) in comparison with the density of 0.5 × 10^6^ and 1.5 × 10^6^ (Figure 12). A significant decrease in proliferation was observed after 7 days of culture at the initial ASC cell count of 0.5 × 10^6^ for PCL (^∗^ *p* < 0.05) and PCL + 5% TCP (^∗∗∗^ *p* < 0.001) compared to 0.9 × 10^6^ and after 7 days of culture on PCL + 5% TCP for 1.5 × 10^6^ (*p* < 0.05). Interestingly, the differences between the density 0.9 × 10^6^ and 1.5 × 10^6^ were not significant after 21 days of cultivation, while they were observed comparing the densities of 0.5 × 10^6^ and 0.9 × 10^6^ (^∗^ *p* < 0.05). Result of analysis is shown in Supplementary Materials (Tables 5 and 6).

### 4.8. Cellular Distribution

**Figure 13:**
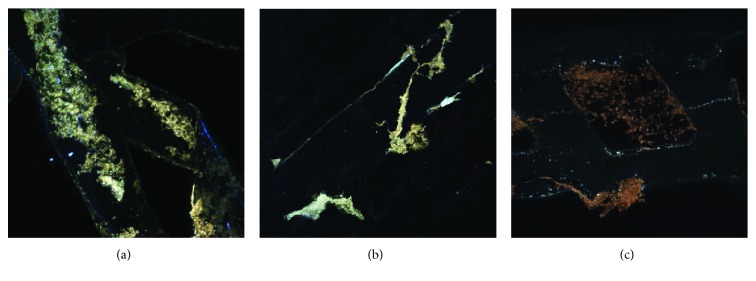
The representative images of human adipose stem cells, seeding into PCL after 21 days of culture. Seeding density of ASC: 0.9 × 10^6^/scaffold. Representative images are shown at 10x magnification. Scale bars represent 100 *μ*m. Images were taken by using a Nikon Eclipse Ti confocal microscope. Representative images are from one donor.

**Figure 14 fig14:**
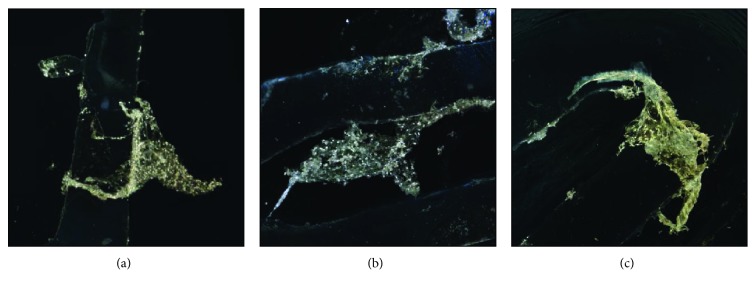
The representative images of human adipose stem cells seeding into PCL + 5% TCP after 21 days of culture. Seeding density of ASC: 0.9 × 10^6^/scaffold. Representative images are shown at 10x magnification. Scale bars represent 100 *μ*m. Images were taken by using a Nikon Eclipse Ti confocal microscope. Representative images are from one donor.

**Figure 15 fig15:**
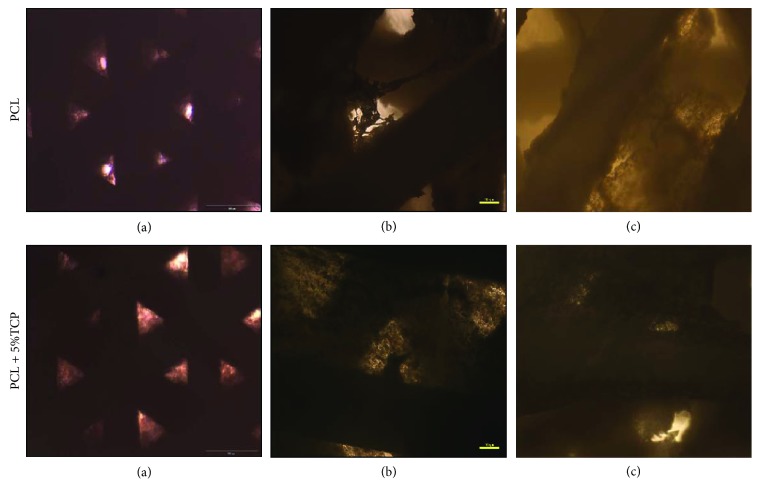
The representative images of human adipose stem cells seeding into PCL and PCL + 5% TCP after 42 days of culture. Seeding density of ASC: 0.9 × 10^6^/scaffold. Representative images are shown at 10x magnification. Scale bars represent 100 *μ*m. Images were taken by using an Olympus CKX41 microscope. (a) Proximal part of seeding scaffolds. (b, c) Distal part of seeding scaffolds. Representative images are from one donor.

**Figure 16 fig16:**
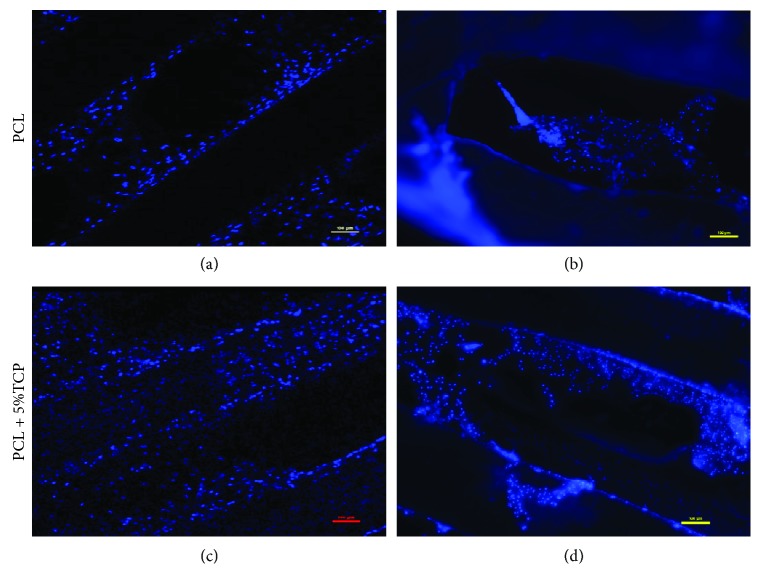
Fluorescence microscope images for DAPI staining (blue nucleus) of human adipose stem cells seeded onto the PCL and PCL + 5% TCP after 21 days of culture. (a, c) Single cell nucleus of ASC. (b, d) Clusters of nucleus inside scaffolds. Seeding density = 0.9 × 10^6^. Representative images are shown at 10x magnification. Scale bars represent 100 *μ*m. Representative images are from one donor.

**Figure 17 fig17:**
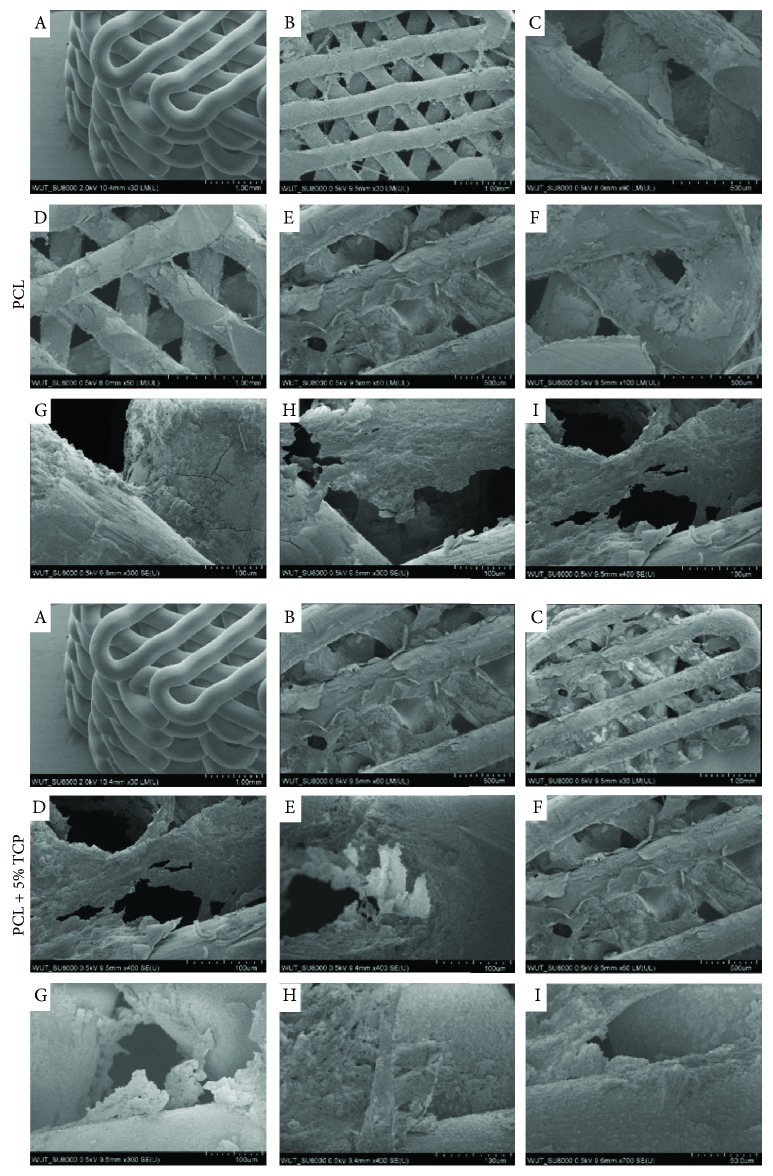
SEM analyses of PCL and PCL + 5% TCP composite after 42 days of ASC culture. Density seeding 0.9 × 10^6^. (a) Control PCL without ASCs. (b, c) Proximal part of scaffolds. (d–i) Distal part of scaffolds. Representative images are from one donor.

**Figure 18:**
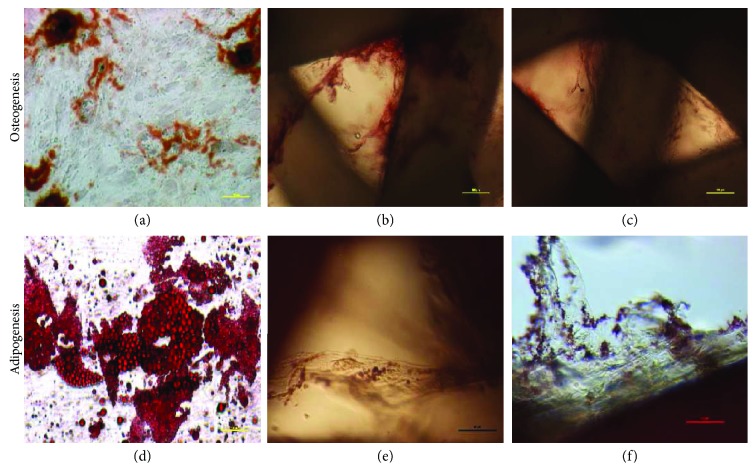
The representative images of human adipose stem cells seeding into PCL and PCL + 5% TCP after 21 days of culture. (a) Osteogenic differentiation of ASCs in 2D culture (density: 0.3 × 10^6^). (b) Osteogenic differentiation (ARS) in PCL scaffold. (c) Osteogenic differentiation in PCL + 5% TCP. (d) Adipogenic differentiation (Oil Red O) of ASCs in 2D culture. (e) Adipogenic differentiation in PCL scaffold. (f) Adipogenic differentiation in PCL + 5% TCP scaffold. Seeding density of ASCs: 0.9 × 10^6^ per scaffold. Representative images are shown at 10x magnification. Scale bars represent 100 *μ*m. Images were taken by using an Olympus CKX41 microscope.

Analysis of images of human ASC cell seeding onto PCL scaffold shows high adhesion capacity (Figures 13(a)–13(c)) and cell migration (Figures 13 [Fig fig14] [Fig fig15] [Fig fig16] [Fig fig17]–18). Both types of scaffolds promote adhesion and proliferation of cells (Figure 15). Microscopic analysis of fibers of scaffolds (Figures 13 and 14) shows that ASCs adhere to scaffold surface and make extensive cell clusters. Cell distribution was homogeneous throughout the scaffold material (Figure 15). DAPI staining was used to evaluate the proliferation rate of adipose stem cells seeded on PCL and PCL + 5% TCP cultured up to 21 days. We observed that seeded ASCs adhere to the surface and migrate into the scaffolds (Figure 16).

SEM images of the PCL and PCL + 5% TCP scaffold surface and cross section were collected at different magnifications. The cells are evenly distributed over its entire porous surface of scaffolds (Figure 17). Also, at the fiber interface, the cells adhering the scaffold are visible (Figures 17(c), 17(d), and 17(g)). In the proximal part of the scaffold, attached layers of cells were observed (Figures 17(e), 17(f), 17(h), and 17(i)). Likewise, in the distal part, the adherent cell-forming groups cover the surface. The cells are characterized by the presence of many cell convexities (Figures 17(f), 17(g), and 17(i)). In all cases of the tested PCL and PCL + 5% TCP scaffold surfaces, positive ASC cell responses were noted.

### 4.9. Osteogenic Potential of ASCs into the PCL and PCL Covered with 5% Tricalcium Phosphate

**Figure 19:**
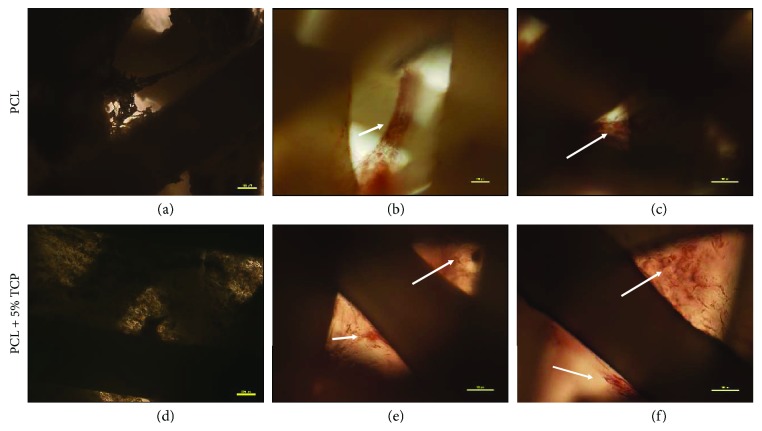
The representative images of human adipose stem cells seeding into PCL and PCL + 5% TCP after 42 days of culture. (a, d) Control, undifferentiated of ASCs. (b, c, e, f) Osteogenic differentiation (ARS). Seeding density of ASC: 0.9 × 10^6^/scaffold. Representative images are shown at 10x magnification. Scale bars represent 100 *μ*m. Images were taken by using an Olympus CKX41 microscope.

Alizarin Red S staining was employed to observe the calcium deposition in the osteogenic differentiation of ASCs on PCL and PCL + 5% TCP scaffolds for 7 and 14 days postdifferentiation. Alizarin Red-positive nodules formed in ASCs on both scaffolds uniformly (Figures 18(a)–18(c) and 19). Adipogenic differentiation of the cells into PCL and PCL + 5% TCP showed accumulated intracellular lipid droplet as revealed by Oil Red O staining (Figures 18(d)–18(f)).

**Figure 20:**
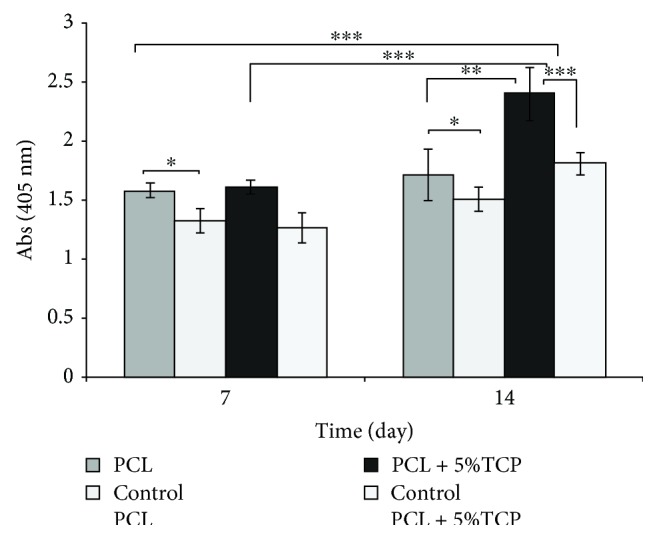
Alkaline phosphate activity of cultured osteoblasts on PCL and PCL + 5% TCP scaffolds after 7 and 14 days. Control: undifferentiated of ASCs. Statistical analysis: ^∗^ *p* < 0.05, ^∗∗^ *p* < 0.01, and ^∗∗∗^ *p* < 0.001.

The osteogenic assay showed that cell-seeded PLC + 5% TCP scaffolds cultured in the osteogenic medium presented significantly greater signal of staining dye and higher alkaline phosphatase level production during the 14-day culture period (^∗∗^ *p* < 0.01), compared with the control medium and PCL scaffold. These results imply that the modification of scaffolds may influence the degree of ASC cell differentiation (Figure 20). See raw data in Supplementary Materials (Table 7).

## 5. Discussion

In the present study, ASCs were obtained from abdomen tissue from 17 female donors over a broad age range (49 ± 6 years). Analysis indicated that the overall yield of SVF cells was 0.36 × 10^6^ per milliliter of lipoaspirate. Our results are comparable with data published by Suga et al. [17], Millan et al. [18], Markarian et al. [19], Condé-Green et al. [20], and Aronowitz et al. [21]. Nevertheless, there are reports in the literature that indicate a significantly higher number of SVFs obtained from 2–6 × 10^6^/ml of adipose tissue [22], but also it is obtained from 0.5 × 10^4^ to 7.95 × 10^5^ per 1 g of adipose tissue [19, 23–25]. The reason for such individual variability of ASCs is not fully understood yet. One of the causes can be lack of standard isolation and culture procedures. As a result, comparison and interpretation of the various scientific researches are restricted. Standardization of these parameters may increase reliability and repeatability of results, but there are also factors affecting the quality of ASC, which cannot be standardized, i.e., age, ethnicity, medical history, and BMI index [5, 23, 26–29].

There are many conflicting reports about the effect of donor's age and the number of cells obtained from adipose tissue. It is believed that cells residing in the elderly are subjected to age-related changes and thus contribute less to tissue rejuvenation [30]. In contrast, some studies reported that donor age does not affect the characteristics, proliferation, or osteogenic differentiation potential of ASCs allowing expansion of the use of multipotent mesenchymal stem cell donors [12, 31]. Our results demonstrated no effect of donor age (49 ± 6 years) on the cell expansion and differentiation potential. Discrepancies of many reports may imply from the broad age ranges and the health status of the donors that were studied.

The kinetics of the proliferation of cells obtained from human adipose tissue shows that the highest number of ASCs can be achieved in passage 5 and after passage 16, proliferation is significantly decrease. This may suggest that ASCs can be cultured *in vitro* for a long time without impairing their proliferative capacity [32]. In contrast, some studies have reported decrease of ASC proliferation capacity in number of passages [33–36]. On the other hand, we also observed large deviation of the mean results after 5^th^ passage. These differences can be due to a noise measurement or differences depending on the donors. The results obtained thus suggest that age-related changes in ASC number should be taken individually into account whenever these cells are considered for clinical applications. Interestingly, we also observed that the percentage of colony-forming units from ASCs was significantly higher than results demonstrated by Rodriguez et al. [25] or Aronovitz et al. [24] and slightly lower than the results by Güven et al. [37]. Numerous studies have reported that CFU-F may decrease with increasing age of the donor [38]. Therefore, ASCs may require either a larger amount of adipose tissue or a pretreatment strategy to increase proliferation *in vitro* and the clonogenic ability of ASCs.

In our study, we demonstrated that along with the next passage of ASCs, the accumulation of calcium phosphate in cells *in vitro* marked with ARS is increasing. These changes in the ability to differentiate are confirmed by other reports, where ALP levels and the expression of osteogenic genes began to increase with rising passage [39]. Studies have also reported that after intensive passage of human ASC cells, osteogenic differentiation begins to prevail and adipogenic potential disappears [35, 40]. Some reports that osteogenic potential decreases during aging [6, 14, 25, 33, 41] while others demonstrate that human adipose tissues have high osteogenic potential and are most important in response to osteogenic factors [42]. The result we obtained suggests that the use of conditioned cells of older passage may give better effects of osteogenic differentiation than the use of early passage cells in bone regeneration [43–45].

It is well known in fact that surface topography strongly affects implant performance and influences cell adhesion, cell shape, and tissue organization as well as the production of local microenvironment [46, 47]. In the present study, microscopic observations noted that cells can migrate along the whole scaffold and the pore size range used is not a limitation for cell migration. It was observed that the cells on PCL scaffolds are distributed in small amount heterogeneously across the entire area of the scaffold, while on PCL + 5% TCP constitute a compact cell structure inside the scaffold. It was also noted that after 42 days of culture, many cells filled the space between the scaffolding fibers, which additionally suggests that the cells need more time in seeding the scaffold structure.

Seeding of cells on the scaffolds is a critical step in the process for the construction of polymer-cell scaffolds and often determines the quality of tissue engineering products. The criteria for an optimal seeding method assume that cell distribution should be homogeneous on scaffolds, with high efficiency and easy to use with minimal cell damage during the procedure [48]. We observed that the increase in the volume of cell suspension is a significant parameter of the reduction in the efficiency of seeding scaffolds. The highest efficiency of seeding ASCs on scaffolds was obtained with seeding volume of 35 *μ*l and the lowest using 50 *μ*l. Similar results were presented by Zhou et al. [49] and Buckley and O'Kelly [50], who observed a tendency that, depending on the volume of the cell suspension, the efficiency of seeding decreases. Buckley and O'Kelly [50] demonstrated that the maximum yield was obtained using a volume of 25 *μ*l (85.4% ± 4.9%) and the smallest yield using a volume of 50 *μ*l (67.7% ± 2.2%) and 100 *μ*l (43.8% ± 3, 2%).

Comparing methods of seeding scaffolds, we observed the effect of cell aspiration into PCL and PCL + 5% TCP when the method of saturation of cell suspension with additional incubation was used. Literature reports show that aspiration of cell suspension and capillary force is of great importance in the organization of cells and seeding into scaffolds [51]. Therefore, in our presented methods of the seeding process, we used an additional incubation step. This modification contributed to a better cell adhesion onto scaffolds and effective cell migration inside porous. Increased seeding efficiency was observed when using the saturation of cell suspension into scaffolds with additional incubation. Basic static cell seeding method was not generating this level of seeding efficiency. Furthermore, we also observed that the cell seeding efficiency did differ significantly between scaffold materials and cell seeding methods (^∗∗∗^ *p* < 0.001). These may imply from the increased hydrophilicity of the polymer, as well as from the presence of the calcium phosphate component in the scaffold matrix, which can increase the absorption of proteins [48, 49, 52–54]. These suggest that the architecture and modification of scaffolds may affect cell distribution during seeding, as well as active migration and tissue formation. Furthermore, using the method of saturation of cell suspension increases significantly the cell seeding on PCL + 5% TCP after 7 days of culture.

Obtaining of the optimal density of seeding ASC cells of scaffolds tested is difficult due to the complicated structure of the scaffolds and many interactions between cell scaffolds. The results of these studies suggest that optimal ASC growth on scaffolds was observed at the initial density of 0.9 × 10^6^ to the surface of scaffold, after 21 days of culture. Interestingly, the differences between the density 0.9 × 10^6^ and 1.5 × 10^6^ were not significant after 21 days of culture and significant between 0.5 × 10^6^ and 0.9 × 10^6^ cell seeding densities (^∗^ *p* < 0.05). Our result are similar with other previous studies and suggest that time can significantly influence the seeding efficiency [55, 56]. On the other hand, the positive effect of using low cell densities for seeding scaffolds was noted by Zhou et al. [49]. In contrast, Tan et al. [57] showed that the higher number of cell seeding increased the adhered cells on the scaffolds. Thevenot et al. [47] noted that when a static method was used, 25% of cell seeding on a scaffold underwent apoptosis after 3 hours of seeding and by a dynamic seeding method, over 50% of cell seeding underwent apoptosis. These observations may imply that the time and method of seeding may be more important than the initial cell density for seeding and the use of a large number of cells does not necessarily mean better adhesion and more efficient seeding. These results confirm with the literature data, in which cell attachment was low on scaffolds on initial seeding (determined at 15%) and, depending on the pore size, could decrease even up to 40% after 4 days of culture and then can gradually increase after 21 days of culture [52].

Evaluation of an osteogenic marker ALP of ASCs revealed that changes in the scaffold structures could influence the degree of cell differentiation. A significant increase in osteogenesis of ASCs on the PCL + 5% TCP scaffold was observed after 14 days of culturing, compared to undifferentiated ASCs cultured in a standard culture medium. The results obtained thus are consistent with those of the previous studies reporting an increase in ALP level activity after 14 days of ASC differentiation on scaffolds [40, 58–60]. In addition, studies have reported that there are reports that show that the addition of tricalcium phosphate may improve the ability of cells to differentiate towards osteogenesis in PCL scaffold [56, 61, 62]. In contrast, there are also studies reported that an admixture of 3–5% TCP or HA does not show yield significant differences in the cell mineralization on scaffolds [59, 63].

## 6. Conclusions

The conducted experiments show that the tested materials have a positive influence on ASC cell adhesion and proliferation and applied material may play a role as a temporary extracellular matrix. PCL and PCL + 5% TCP are promising cellular platforms for regenerative therapy for further *in vitro* and *in vivo* studies. Porous PCL scaffold with 5% TCP can play an important role in cell migration, adhesion and infusion of nutrients, promoting proliferation, and differentiation of ASC cells for osteogenesis better than PCL. ASC cells adhere to scaffold surfaces; for better seeding efficiency, cells should be cultured for a longer time. From the perspective on the potential use for clinical applications, PCL covered with 5% tricalcium phosphate may contribute to the promotion of bone repair and provide an appropriate model to support the regeneration of new bone for future tissue engineering strategy.

## Data Availability

The numeric and graphic data used to support the findings of this study are included within the Supplementary Materials' file.

## Supplementary Materials

Supplementary Materials MTS standard curve. Table 1: characterization of donors and number of SVF (*n* = 17). Table 2: growth kinetics of human ASCs. Summary table of average number of human ASCs. *M* ± SD (*n* = 3). Table 3: seeding efficiency of human ASCs within PCL and PCL + 5% TCP scaffolds. Summary table of absorbance values measured by the MTS test for seeding hASC into PCL and PCL + 5% TCP. *M* ± SD (*n* = 3). Table 4: summary table of absorbance values measured by the MTS test for seeding human ASC into PCL and PCL + 5% TCP. *M* ± SD (*n* = 6). Table 5: summary table of absorbance values measured by the MTS test for seeding human ASC into PCL and average number of cells, measured by the standard curve of MTS. *M* ± SD (*n* = 3). Table 6: summary table of absorbance values measured by the MTS test for seeding human ASC into PCL + 5% TCP, and average number of cells, measured by the standard curve of MTS. *M* ± SD (*n* = 3). Table 7: summary table of absorbance values measured by the ALP test for seeding human ASC into PCL + 5% TCP. *M* ± SD (*n* = 5).
