# Supplementary material for: Characterization and Optimization of the Seeding Process of Adipose Stem Cells on the Polycaprolactone Scaffolds
Source: Stem Cells Int. 2019 Feb 20;2019:1201927. doi: 10.1155/2019/1201927 (PMC6402208; doi:10.1155/2019/1201927)
Supplement: Supplementary Materials — MTS standard curve. Table 1: characterization of donors and number of SVF (n = 17). Table 2: growth kinetics of human ASCs. Summary table of average number of human ASCs. M ± SD (n = 3). Table 3: seeding efficiency of human ASCs within PCL and PCL + 5% TCP scaffolds. Summary table of absorbance values measured by the MTS test for seeding hASC into PCL and PCL + 5% TCP. M ± SD (n = 3). Table 4: summary table of absorbance values measured by the MTS test for seeding human ASC into PCL and PCL + 5% TCP. M ± SD (n = 6). Table 5: summary table of absorbance values measured by the MTS test for seeding human ASC into PCL and average number of cells, measured by the standard curve of MTS. M ± SD (n = 3). Table 6: summary table of absorbance values measured by the MTS test for seeding human ASC into PCL + 5% TCP, and average number of cells, measured by the standard curve of MTS. M ± SD (n = 3). Table 7: summary table of absorbance values measured by the ALP test for seeding human ASC into PCL + 5% TCP. M ± SD (n = 5). [file 1201927.f1.pdf]

SUPPLEMENTARY MATERIALS (Manuscript No 1201927).

MTS standard curve.

Effect of cell number on absorbance at 490 nm. Various numbers of ASCs were cultured in a final volume of 200 µl/well by incubating at 37°C. MTS reagent was added and absorbance at 490nm was recorded using spectrophotometer (Multiscan Go, Thermoscientific, Vantaa, Finland) plate reader. Each point represents a mean of 3 replicates. Assay was performed according to the kit protocol.

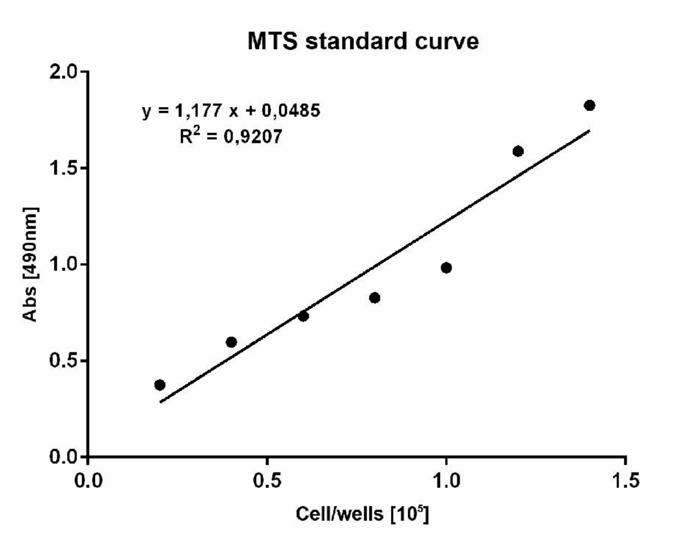

TABLE 1: Characterization of donors and number of SVF (n=17).

Donor (female)	Age [years]	Volume of lipoaspirate [ml]	Viability of SVF [%]	Total cell number (SVF) [x10^6]
1	40	200	84	83.6
2	52	184	90	35.8
3	59	180	92	103.0
4	43	200	80	97.0
5	46	160	92	101.0
6	47	160	98	53.0
7	52	180	91	152.0
8	51	180	85	55.80
9	41	180	92	30.0
10	43	110	80	30.40
11	47	105	80	66.0
12	55	120	77	27.0
13	41	200	80	35.0
14	56	210	80	36.0
15	50	90	88	28.0
16	55	160	88	66.0
17	57	160	88	20.0

TABLE 2: Growth kinetics of human ASCs. Summary table of average number of human ASCs. M ± SD. (n=3).

Passage	Average number of cells [10^6]. M± SD	Passage	Average numer of cells [10^6]. M± SD
**0**	0.53 ± 0.152	**12**	1.01 ± 0.175
**1**	0.97 ± 0.075	**13**	1.01 ± 0.125
**2**	1.53 ± 0.212	**14**	0.62 ± 0.037
**3**	2.53 ± 0.351	**15**	1.50 ± 0.173
**4**	1.83 ± 0.321	**16**	1.46 ± 0.152
**5**	3.09 ± 0.062	**17**	0.68 ± 0.076
**6**	1.31 ± 0.331	**18**	0.80 ± 0.173
**7**	0.99 ± 0.228	**19**	0.41 ± 0.036
**8**	0.82 ± 0.175	**20**	0.28 ± 0.026
**9**	1.21 ± 0.314	**21**	0.26 ± 0.028
**10**	1.31 ± 0.220	**22**	0.13 ± 0.026
**11**	1.63 ± 0.413	**23**	0.12 ± 0.037

TABLE 3:Seeding efficiency of human ASCs within PCL and PCL+5%TCP scaffolds. Summary table of absorbance values measured by the MTS test for seeding hASC into PCL and PCL+5%TCP. M ± SD, (n=3). hASC- human adipose stem cells, PCL-polycaprolactone, PCL+5% TCP polycaprolactone covered 5% tricalcium phosphate.

		**hASC PCL**				**hASC PCL +5% TCP**			
**50 µl**	**Time [day]**	**1**	**3**	**7**	**21**	**1**	**3**	**7**	**21**
	**Abs [490nm]**	0.57	0.65	0.61	0.71	0.46	0.56	0.60	0.65
		0.58	0.75	0.63	0.64	0.48	0.59	0.36	0.44
		0.59	0.76	0.68	0.69	0.38	0.46	0.53	0.67
	**M**	**0.58**	**0.72**	**0.64**	**0.68**	**0.44**	**0.53**	**0.49**	**0.58**
	**SD**	**0.05**	**0.04**	**0.12**	**0.12**	**0.05**	**0.06**	**0.12**	**0.12**
	**Time [day]**	**1**	**3**	**7**	**21**	**1**	**3**	**7**	**21**
**35 µl**	**Abs [490nm]**	0.47	0.80	0.83	0.97	0.82	0.84	0.92	1.25
		0.48	0.65	0.75	0.87	0.61	0.89	0.89	1.22
		0.37	0.77	0.89	0.78	0.60	0.83	0.79	1.00
	**M**	**0.44**	**0.74**	**0.82**	**0.87**	**0.67**	**0.85**	**0.87**	**1.15**
	**SD**	**0.06**	**0.08**	**0.06**	**0.09**	**0.12**	**0.03**	**0.06**	**0.13**

TABLE 4: Summary table of absorbance values measured by the MTS test for seeding human ASC into PCL and PCL+5%TCP. M ± SD, (n=6).

**Seeding methods**	**the basic static cell seeding into PCL [Abs=490nm]**		**the basic static cell seeding into PCL+5%TCP [Abs=490nm]**		**the saturation of cell suspension with incubation into PCL [Abs=490nm]**		**the saturation of cell suspension with incubation into PCL+5%TCP [Abs=490nm]**	
Time [day]	1	7	1	7	1	7	1	7
	0.565	0.797	0.259	0.631	0.210	1.254	0.818	1.431
	0.623	0.793	0.384	0.641	0.198	1.428	1.048	1.774
	0.544	0.7320	0.352	0.594	0.287	1.012	0.954	1.333
	0.495	0.759	0.298	0.614	0.394	1.125	1.067	1.568
	0.619	0.759	0.391	0.659	0.378	0.987	0.924	2.116

	0.583	0.721	0.341	0.710	0.417	1.102	1.323	1.581
**M**	**0.571**	**0.760**	**0.337**	**0.641**	**0.314**	**1.151**	**1.023**	**1.633**
**SD**	**0.048**	**0.031**	**0.050**	**0.040**	**0.096**	**0.165**	**0.172**	**0.279**

TABLE 5: Summary table of absorbance values measured by the MTS test for seeding human ASC into PCL. and average number of cells. measured by the standard curve of MTS. M ± SD, (n=3).

**Time [day]**	**3**	**7**	**21**
**Initial density**	**Abs MTS [490nm]**		
0.5 $x10^6$	0.926 ± 0.108	0.861 ± 0.139	1.586 ± 0.081
0.9 $x10^6$	1.135 ± 0.084	1.462 ± 0.095	1.060 ± 0.168
1.5 $x10^6$	1.159 ± 0.104	1.06 ± 0.109	1.792 ± 0.173
**Initial density**	**Average number of cells [$x10^5$] after calculate of the standard curve MTS [$x10^5$]**		
0.5 $x10^6$	0.746 ± 0.04	0.860 ± 0.077	1.580 ± 0.028
0.9 $x10^6$	0.923 ± 0.030	1.731 ± 0.040	1.991 ± 0.101
1.5 $x10^6$	1.159 ± 0.047	1.061 ± 0.051	1.187 ± 0.105

TABLE 6: Summary table of absorbance values measured by the MTS test for seeding human ASC into PCL+5% TCP . and average number of cells. measured by the standard curve of MTS (M ± SD. (n=3).

**Time [day]**	**3**	**7**	**21**
**Initial density**	**Abs MTS [490nm]**		
0.5 $x10^6$	0.965 ± 0.061	0.751 ± 0.113	1.715± 0.060
0.9 $x10^6$	1.155 ± 0.093	2.067 ± 0.229	2.381 ± 0.060
1.5 $x10^6$	1.567 ± 0.100	1.426 ± 0.120	1.906 ± 0.328
**Initial denisty**	**Average number of cells [$x10^5$] after calculate of the standard curve MTS [$x10^5$]**		
0.5 $x10^6$	0.779 ± 0.011	0.597 ± 0.055	1.480 ± 0.223
0.9 $x10^6$	0.941 ± 0.038	1.715 ± 0.153	2.049 ± 0.109
1.5 $x10^6$	1.291 ± 0.044	1.171 ± 0.061	1.579 ± 0.238

TABLE 7: Summary table of absorbance values measured by the ALP test for seeding human ASC into PCL+5% TCP. (M ± SD. (n=5).

**Type of scaffold**	**PCL**	**PCL control**	**PCL**	**PCL control**	**PCL+5%TCP**	**PCL+5%TCP control**	**PCL+5%TCP**	**PCL+5%TCP control**
**Time [day]**	**7**		**14**		**7**		**14**	
	1.30	1.20	1.40	1.23	1.35	1.23	1.87	1.53
	1.45	1.22	1.38	1.10	1.34	1.32	1.90	1.43
	1.35	1.42	1.47	1.13	1.40	1.30	1.97	1.40
	1.11	1.2	1.40	1.0	1.30	1.20	1.95	1.38
	1.30	1.2	1.41	1.0	1.16	1.21	1.93	1.43
**M**	**1.30**	**1.22**	**1.41**	**1.1**	**1.31**	**1.25**	**1.92**	**1.43**
**SD**	**0.12**	**0.11**	**0.03**	**0.1**	**0.09**	**0.05**	**0.08**	**0.06**
